# MASS cohort: Multicenter, longitudinal, and prospective study of the role of microbiome in severe pneumonia and host susceptibility

**DOI:** 10.1002/imt2.218

**Published:** 2024-06-25

**Authors:** Xin Wei, Li Guo, Hongliu Cai, Silan Gu, Lingling Tang, Yuxin Leng, Minghui Cheng, Guojun He, Yijiao Han, Xindie Ren, Baoyue Lin, Longxian Lv, Huanzhang Shao, Mingqiang Wang, Hongyu Wang, Dan Dang, Shengfeng Wang, Nan Wang, Peng Shen, Qianqian Wang, Yinghe Xu, Yongpo Jiang, Ning Zhang, Xuwei He, Xuntao Deng, Muhua Dai, Lin Zhong, Yonghui Xiong, Yujie Pan, Kankai Tang, Fengqi Liu, Bin Yang, Lili Ren, Jianwei Wang, Chao Jiang, Lingtong Huang

**Affiliations:** ^1^ Life Sciences Institute and Department of Critical Care Medicine of First Affiliated Hospital Zhejiang University Hangzhou China; ^2^ NHC Key Laboratory of Systems Biology of Pathogens and Christophe Mérieux Laboratory, National Institute of Pathogen Biology Chinese Academy of Medical Sciences & Peking Union Medical College Beijing China; ^3^ Department of Critical Care Medicine, The First Affiliated Hospital Zhejiang University School of Medicine Hangzhou China; ^4^ State Key Laboratory for Diagnosis and Treatment of Infectious Diseases, National Clinical Research Center for Infectious Diseases, Collaborative Innovation Center for Diagnosis and Treatment of Infectious Diseases, The First Affiliated Hospital Zhejiang University School of Medicine Hangzhou China; ^5^ Department of Infectious Diseases Shulan (Hangzhou) Hospital Hangzhou China; ^6^ Department of Intensive Care Unit Peking University Third Hospital Beijing China; ^7^ Department of Critical Care Medicine, Henan Key Laboratory for Critical Care Medicine, Zhengzhou Key Laboratory for Critical Care Medicine, Henan Provincial People's Hospital; Zhengzhou University People's Hospital Henan University People's Hospital Zhengzhou China; ^8^ Department of Emergency Intensive Care Unit The Fifth Clinical Medical College of Henan University of Chinese Medicine Zhengzhou China; ^9^ Department of Critical Care Medicine Xi'an People's Hospital (Xi'an No.4 Hospital) Xi'an China; ^10^ Department of Critical Care Medicine The Second Affiliated Hospital of Zhengzhou University Zhengzhou China; ^11^ Department of Critical Care Medicine The First Hospital of Jiaxing Jiaxing China; ^12^ Department of Critical Care Medicine Taizhou Hospital of Zhejiang Province affiliated with Wenzhou Medical University Taizhou China; ^13^ Department of Critical Care Medicine Lishui People's Hospital Lishui China; ^14^ Department of Critical Care Medicine Tongde Hospital of Zhejiang Province Hangzhou China; ^15^ Department of Critical Care Medicine The First People's Hospital of Pinghu Pinghu China; ^16^ Department of Critical Care Medicine Lanxi Hospital of Traditional Chinese Medicine Lanxi China; ^17^ Department of Critical Care Medicine Wenzhou Central Hospital Wenzhou China; ^18^ Department of Critical Care Medicine The First People's Hospital of Huzhou Huzhou China; ^19^ Center for Infectious Diseases Vision Medicals Co., Ltd. Guangzhou Guangdong China

## Abstract

The MASS cohort comprises 2000 ICU patients with severe pneumonia, covering community‐acquired pneumonia, hospital‐acquired pneumonia, and ventilator‐associated pneumonia, sourced from 19 hospitals across 10 cities in three provinces. A wide array of samples including bronchoalveolar lavage fluid, sputum, feces, and whole blood are longitudinally collected throughout patients' ICU stays. The cohort study seeks to uncover the dynamics of lung and gut microbiomes and their associations with severe pneumonia and host susceptibility, integrating deep metagenomics and transcriptomics with detailed clinical data.

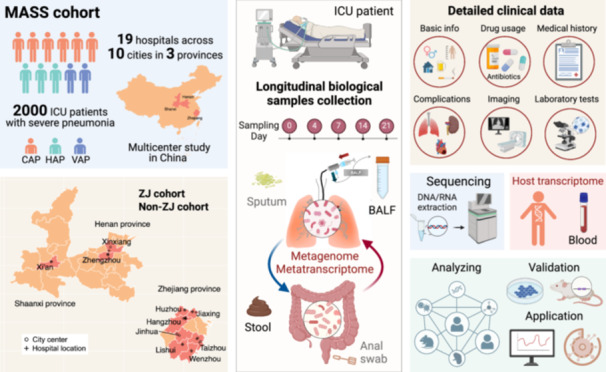

To the Editor,

Severe pneumonia, including community‐acquired pneumonia (CAP), hospital‐acquired pneumonia (HAP), and ventilator‐associated pneumonia (VAP), remains a major challenge in critical care, with high morbidity and mortality [1, 2]. The disruption of lung microbiome, as seen with SARS‐CoV‐2 [3], is associated with disease progression by triggering severe inflammatory responses and influencing immune functions [4], notably through alveolar macrophages, which are crucial for pathogen clearance [5]. The interplay between the lung and gut microbiomes, known as the gut‐lung axis, also plays a critical role in the host's immune response and overall health, especially in intensive care unit (ICU) patients [6, 7].

Dysbiosis in these microbiomes may contribute to severe conditions like pneumonia and acute respiratory distress syndrome (ARDS), impacting patient outcomes and survival [6]. However, understanding the connections between the lung and gut microbiota and host susceptibility in severe pneumonia is still limited, often hindered by small cohort sizes and the lack of comprehensive data from multiple centers [8, 9].

To address these gaps, we introduce the MASS cohort—a multicenter, longitudinal, and prospective study of the role of microbiome in severe pneumonia and host susceptibility. From 2023 to 2025, we plan to collect bronchoalveolar lavage fluid (BALF), sputum, feces, and whole blood from 2000 ICU patients with severe pneumonia, alongside detailed clinical data. Our goal is to uncover the dynamics of lung and gut microbiomes and their relationship to host susceptibility (see “Outcome assessment for host susceptibility” in Supporting Information), as an extension of our previous multi‐center research [10, 11]. In addition, the multicenter observational study may serve as a surveillance tool for emerging respiratory infectious diseases, laying a solid foundation for subsequent validation studies and clinical interventions for severe pneumonia.

## STUDY DESIGN

**Figure 1:**
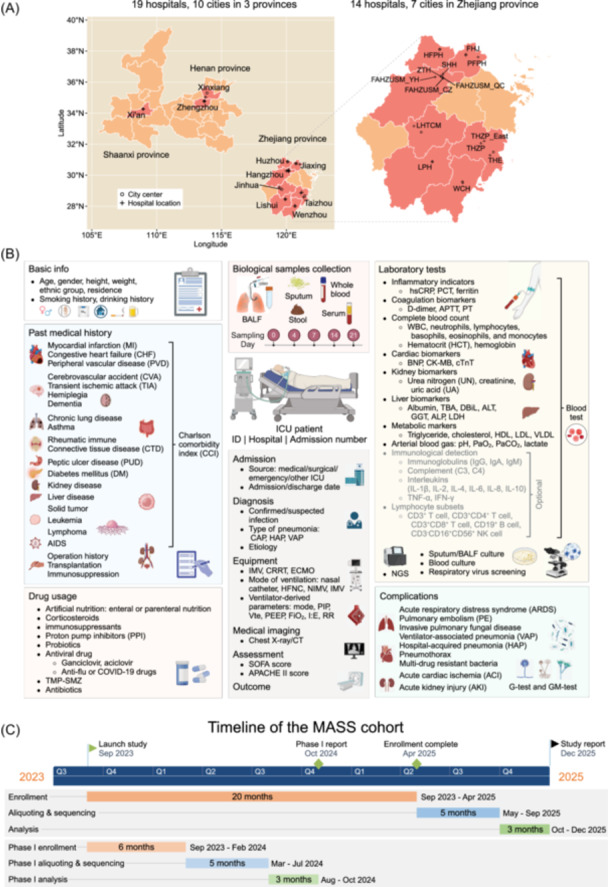
Study design of the MASS cohort. (A) Geographical distributions of the 19 medical centers across 10 cities (red) in three provinces of China. Fourteen centers are located in seven cities of Zhejiang province (zoomed‐in view on the right), four in Henan province, and one in Shaanxi province. (B) Overview of clinical data and biological sample collection. (C) Timeline of MASS cohort.

MASS is designed as a prospective, multicenter cohort comprising 26 ICUs across 19 medical centers, spanning 10 cities of three provinces, with a total of more than 800 ICU beds (Figure 1A and Table S1). These ICUs possess extensive expertise in the diagnosis and treatment of severe pneumonia, equipped with skilled medical staff. Among these medical centers, 14 are situated in seven cities of Zhejiang province, four in two cities of Henan province, and one in Shaanxi province. We plan to utilize patients in Zhejiang province as the discovery cohort (ZJ cohort), while patients from other provinces serve as external validation cohorts (non‐ZJ cohorts). Alternatively, considering the potential geographic variation in microbiomes, we plan to allocate 20% of patients of each province as the respective validation cohort. The clinical trial has received approval from the Ethics Committee of the First Affiliated Hospital of Zhejiang University School of Medicine (ethic ID: IIT20230371B and IIT20230183B‐R1) and registered on ClinicalTrials.gov (NCT06114784).

We plan to recruit 2000 ICU patients for longitudinal sample collection and follow‐up visits (Figure 1B and Table S2). Upon enrollment, their basic information, medical history, and imaging data are documented (see “Collection of clinical data” in Supporting Information). Simultaneously, BALF, sputum, stool, and blood samples are collected for laboratory culture and next‐generation sequencing (NGS), among other analyses (see “Methodologies of biological sample processing” and “Methods of bioinformatics analysis” in Supporting Information). When applicable, these samples will be collected longitudinally at 4, 7, 14, and 21 days after ICU admission, alongside the ventilator parameters recorded on those days. Drug usage will be documented on Days 14 and 28, and complications on Day 28. Additionally, follow‐up visits will be conducted on Day 90 and 365 (Table S2).

The primary aims of the MASS cohort are: (1) investigating the dynamic characteristics of the lung and gut microbiomes in severe pneumonia patients and (2) exploring potential associations between microbiomes and host susceptibility to severe pneumonia.

The secondary aims of the MASS cohort include: (1) examining the relationships between lung microbiome and treatments, drug usage, and exposure; (2) investigating the variations in drug‐resistance genes and horizontal gene transfer (HGT) within the lung and gut microbiomes of severe pneumonia patients; (3) identifying the disparities in host characteristics and microbiomes in CAP, HAP, and VAP; (4) charting the lung virome landscape; (5) predicting the 28‐day mortality for ICU patients based on lung microbiome and (6) dynamic monitoring of lung microbiome to predict secondary bacterial infection.

A timeline of the cohort has been established based on the current progress, detailed in the “Progress of the cohort” section (Figure 1C).

## RECRUITMENT

The cohort commenced recruitment in September 2023 (Figure 1C). At the time of writing the announcement (February 2024), we have successfully recruited 286 patients from 15 hospitals (see “Progress of the cohort”). We aim to enroll up to 2000 ICU patients with severe pneumonia to delineate the infections caused by various pan‐kingdom pathogens. Since a definitive diagnosis of severe pneumonia may not be possible at enrollment, patients with suspected lung infections will also be included (see “Definitions” in Supporting Information). Following enrollment, Dr. Lingtong Huang, the project leader, and the principal investigator at each medical center will independently conduct secondary diagnoses for all patients. In cases of diagnostic disagreement, Dr. Hongliu Cai from the First Affiliated Hospital of Zhejiang University School of Medicine will participate in the final evaluation. All enrolled patients are fully informed and provide signed informed consent, without receiving additional financial compensation. For unconscious patients, the consent form will be signed by his/her next of kin. The inclusion and exclusion criteria are outlined below:

Inclusion criteria: Patients with newly acquired lung infections that meet one of the following criteria: (1) receiving invasive or noninvasive mechanical ventilation for acute respiratory failure, with positive end‐expiratory pressure (PEEP) ≥ 5 cm; (2) receiving high‐flow oxygen therapy with a fraction of inspired oxygen (FiO_2_) ≥ 50% and arterial oxygen partial pressure (PaO_2_) to FiO_2_ ratio <300. Patients with suspected lung infections (see “Definitions” in Supporting Information).

Exclusion criteria: (1) patients with expected length of stay in ICU less than 1 day; (2) hospitalized in other ICUs for more than 7 days before being transferred; (3) pregnant; (4) under 18 years old.

## BIOLOGICAL SAMPLING PROCEDURES

We will collect BALF, sputum or endotracheal aspirate (ETA), stool or rectal swab, whole blood, and serum from patients on Days 1, 4, 7, 14, and 21 after admission, when applicable (Figure 1B and Table S2). Sample collection may occur within a 24‐h window around the planned sampling time. For example, if Day 4 sampling is impractical, collection may be rescheduled for Days 3–5 (Day 4 ± 24 h). To minimize the batch effect of supplies, all participating medical centers will use standardized nucleic acid preservation solutions, sampling tubes, and saline solutions supplied quarterly by the First Affiliated Hospital of Zhejiang University School of Medicine. Given the typical low microbial yield in BALF, each hospital will establish negative environmental and instrument controls. Sample collection will conclude if a patient is discharged or transferred from the ICU. The detailed protocols for the collection of various biological samples and negative controls, along with the rest of the analytical protocols can be found in Supporting Information.

## PROGRESS OF THE COHORT

**Figure 2:**
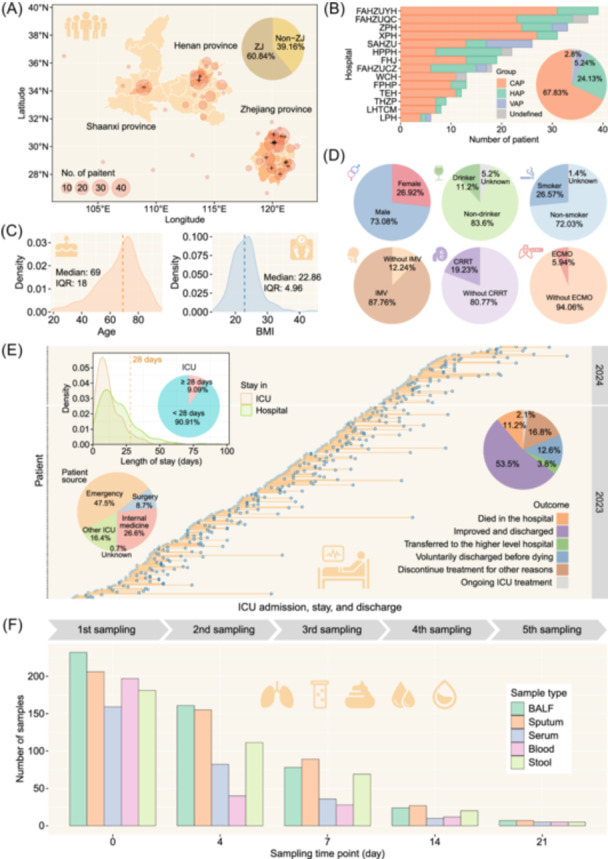
Progress in patient recruitment and biological sampling for the MASS cohort. (A) Geographic distribution of the 286 patients' residences. ZJ represents the patients admitted to medical centers in Zhejiang province, whereas non‐ZJ represents those admitted to medical centers in Shaanxi and Henan provinces. “+” indicate the location of the medical centers. The cities where medical centers are located are painted orange. (B) Number of patients diagnosed with community‐acquired pneumonia, hospital‐acquired pneumonia, and ventilator‐associated pneumonia in each medical center (bar plots) and the overall proportions of different pneumonia (pie chart). The “undefined” group includes four patients with trauma‐associated lung injury and four patients who were considered highly unlikely to have a lung infection. (C) Density plots of patients' age and body mass index. The vertical dashed line denotes the median. (D) The distribution of patients' genders, the proportion of patients having a drinking and smoking history, and the proportion of patients receiving extracorporeal organ support. (E) Distribution of length of stay in intensive care unit (ICU) and patients' sources and outcomes. The light blue dots indicate the time of admission to the ICU, and the blue circles indicate the time of discharge. The “ongoing ICU treatment” group includes hospitalized patients as of April 1, 2024. (F) The number of biological samples collected at five‐time points.

As of February 2024, the MASS cohort had enrolled 286 patients following the inclusion and exclusion criteria. Of these, 174 (60.84%) were from the ZJ cohort and 112 (39.16%) from the non‐ZJ cohorts (Figure 2A). The cohort consisted of 194 (67.83%) patients with CAP, including 22 with stroke‐associated pneumonia, 69 (24.13%) with HAP, and 15 (5.24%) with VAP (Figure 2B). In addition, four (1.4%) patients had trauma‐associated lung injury, while four (1.4%) patients were unlikely to have a lung infection. The median age of enrolled patients was 69 (interquartile range [IQR]: 18), with a median body mass index of 22.86 (IQR: 4.96). The cohort was predominantly male (209 patients; 73.08%), with 32 (11.2%) reporting a history of alcohol use and 76 (26.57%) reporting a history of smoking (Figure 2C,D). In terms of extracorporeal organ support, 251 (87.76%) patients required invasive mechanical ventilation (IMV), 55 (19.23%) received continuous renal replacement therapy (CRRT), and 17 (5.94%) underwent ECMO therapy (Figure 2D).

Among the 286 patients, 202 (70.6%) cases were admitted to ICU on the same day of hospital admission. 84 (29.4%) cases were admitted to the ICU after an average of 7.7 days of hospitalization. A total of 136 (47.5%) were transferred from the emergency department, 76 (26.6%) from internal medicine, 47 (16.4%) from other ICUs, and 25 (8.7%) from surgery (Figure 2E). Outcomes as of April 1, 2024, included 153 (53.5%) patients discharged with improvements, 32 (11.2%) in‐hospital deaths, 11 (3.8%) transfers to higher‐level hospitals, and six (2.1%) in‐hospital patients remaining. Following certain cultural practices in China, 36 (12.6%) patients were discharged voluntarily before death, and 48 (16.8%) patients—or their families—opted for discontinuing treatment or pursuing palliative care. Additionally, 26 (9.09%) patients had extended ICU stays beyond 28 days (Figure 2E).

By the end of February 2024, the cohort had amassed 502 BALF, 484 sputum, 386 stool, 282 whole blood, and 292 serum samples (Figure 2F). Concurrently, 30 negative control samples from each batch of consumables and saline solutions were also collected. The BALF specimens were sent to the National Institute of Pathogen Biology, CAMS & PUMC for aliquoting, using phosphate‐buffered saline as a negative control during this process.

## DISCUSSION

This study aims to utilize a multicenter, large‐cohort, longitudinal approach to robustly characterize the severe pneumonia microbiome across Chinese ICU settings, integrating deep sequencing and advanced analytics to explore the lung microbiome's diversity and its role in respiratory diseases.

The study's limitations include the potential lack of generalizability beyond Chinese ICU patients. The absence of a nonsevere pneumonia control group restricts comparative analyses. An ongoing healthy respiratory virome project, led by Professor Chao Jiang, can provide a healthy control for this study (supported by the National Natural Science Foundation of China, grant no. 82341109). The study's observational nature prevents establishing direct causality between microbiomes and severe pneumonia outcomes. Future studies should include diverse demographics [12], control groups, and experimental validation. Antibiotic use and varying comorbidity and treatment profiles present additional confounding factors, although extensive data collection and propensity score matching may mitigate these effects. Additionally, given the limited research on how environmental factors affect severe pneumonia [13, 14], we plan to incorporate exposome monitoring into the MASS cohort to deepen our understanding [15, 16, 17].

## CONCLUSION

In conclusion, the MASS cohort establishes a rich resource for exploring the microbiome's influence on severe pneumonia and host susceptibility, integrating metagenomic, transcriptomic, and phenomic analyses. This cohort will significantly enhance our understanding of emerging pathogens, risk factors for poor prognosis, and determinants of survival among ICU patients.

## AUTHOR CONTRIBUTIONS

Lingtong Huang, Chao Jiang, Lili Ren, Jianwei Wang, and Lingling Tang designed the trials. Xin Wei, Li Guo, Hongliu Cai, and Silan Gu wrote the study plan. Guojun He, Longxian Lv, Mingqiang Wang, Hongyu Wang, Dan Dang, Shengfeng Wang, Peng Shen, Qianqian Wang, Yinghe Xu, Yongpo Jiang, Xuwei He, Lin Zhong, Yonghui Xiong, Kankai Tang, Fengqi Liu, Yuxin Leng, Minghui Cheng, Yijiao Han, Xindie Ren, Baoyue Lin, Huanzhang Shao, Nan Wang, Ning Zhang, Xuntao Deng, Muhua Dai, Yujie Pan, and Bin Yang participated in the discussion and patient enrollment. All authors have read the final manuscript and approved it for publication.

## CONFLICT OF INTEREST STATEMENT

The authors declare no conflict of interest.

## ETHICS STATEMENT

The study has been approved by the ethics committees of Zhejiang University School of Medicine First Affiliated Hospital (IIT20230371B and IIT20230183B‐R1) and other participating hospitals.

## Supporting information


**File S1.** Supplementary information.

**Table S1.** Medical centers and research institutions participating in MASS cohort. Table S2. Sampling timelines for clinical data and biological samples.

## Data Availability

The data that support the findings of this study are available on request from the corresponding author. The data are not publicly available due to privacy or ethical restrictions. No new data and scripts were generated in this study. Supporting Information: (methods, tables, scripts, graphical abstract, slides, videos, Chinese translated version, and updated materials) may be found in the online DOI or iMeta Science http://www.imeta.science/.
