# Supplementary material for: MASS cohort: Multicenter, longitudinal, and prospective study of the role of microbiome in severe pneumonia and host susceptibility
Source: Imeta. 2024 Jun 25;3(4):e218. doi: 10.1002/imt2.218 (PMC11316923; doi:10.1002/imt2.218)
Supplement: Supplementary file 1 — File S1. Supplementary information. [file IMT2-3-e218-s001.docx]

Supporting information to:

MASS cohort: Multicenter, longitudinal, and prospective study of the role of microbiome in severe pneumonia and host susceptibility

**Running title**: MASS cohort: investigating microbiome's impact on severe pneumonia

Xin Wei ^1#^, Li Guo ^2#^, Hongliu Cai ^3#^, Silan Gu ^4#^, Lingling Tang ^5#^, Yuxin Leng ^6#^, Minghui Cheng ^1^, Guojun He ^3^, Yijiao Han ^3^, Xindie Ren ^3^, Baoyue Lin ^3^, Longxian Lv ^4^, Huanzhang Shao ^7^, Mingqiang Wang ^7^, Hongyu Wang ^8^, Dan Dang ^9^, Shengfeng Wang ^10^, Nan Wang ^10^, Peng Shen ^11^, Qianqian Wang ^11^, Yinghe Xu ^12^, Yongpo Jiang ^12^, Ning Zhang ^13^, Xuwei He ^13^, Xuntao Deng ^13^, Muhua Dai ^14^, Lin Zhong ^15^, Yonghui Xiong ^16^, Yujie Pan ^17^, Kankai Tang ^18^, Fengqi Liu ^18^, Bin Yang ^19^, Lili Ren ^2*^, Jianwei Wang ^2*^, Chao Jiang ^1*^, Lingtong Huang ^1*^

^1^ Life Sciences Institute and Department of Critical Care Medicine of First Affiliated Hospital, Zhejiang University, Hangzhou, China

^2^ NHC Key Laboratory of Systems Biology of Pathogens and Christophe Mérieux Laboratory, National Institute of Pathogen Biology, Chinese Academy of Medical Sciences & Peking Union Medical College, Beijing, China

^3^ Department of Critical Care Medicine, The First Affiliated Hospital, Zhejiang University School of Medicine, Hangzhou, China

^4^ State Key Laboratory for Diagnosis and Treatment of Infectious Diseases, National Clinical Research Center for Infectious Diseases, Collaborative Innovation Center for Diagnosis and Treatment of Infectious Diseases, The First Affiliated Hospital, Zhejiang University School of Medicine, Hangzhou, China

^5^ Department of Infectious Diseases, Shulan (Hangzhou) Hospital, Hangzhou, China

^6^ Department of Intensive Care Unit, Peking University Third Hospital, Beijing, China

^7^ Department of Critical Care Medicine, Henan Key Laboratory for Critical Care Medicine, Zhengzhou Key Laboratory for Critical Care Medicine, Henan Provincial People's Hospital; Zhengzhou University People's Hospital, Henan University People's Hospital, Zhengzhou, China

^8^ Department of Emergency Intensive Care Unit, The Fifth Clinical Medical College of Henan University of Chinese Medicine, Zhengzhou, China

^9^ Department of Critical Care Medicine, Xi’an People's Hospital (Xi’an No.4 Hospital), Xi’an, China

^10^ Department of Critical Care Medicine, The Second Affiliated Hospital of Zhengzhou University, Zhengzhou, China

^11^ Department of Critical Care Medicine, The First Hospital of Jiaxing, Jiaxing, China

^12^ Department of Critical Care Medicine, Taizhou Hospital of Zhejiang Province affiliated with Wenzhou Medical University, Taizhou, China

^13^ Department of Critical Care Medicine, Lishui People's Hospital, Lishui, China

^14^ Department of Critical Care Medicine, Tongde Hospital of Zhejiang Province, Hangzhou, China

^15^ Department of Critical Care Medicine, The First People’s Hospital of Pinghu, Pinghu, China

^16^ Department of Critical Care Medicine, Lanxi Hospital of Traditional Chinese Medicine, Lanxi, China

^17^ Department of Critical Care Medicine, Wenzhou Central Hospital, Wenzhou, China

^18^ Department of Critical Care Medicine, The First People’s Hospital of Huzhou, Huzhou, China

^19^ Center for Infectious Diseases, Vision Medicals Co., Ltd, Guangzhou, Guangdong, China

^#^ These authors contributed equally: Xin Wei, Li Guo, Hongliu Cai, Silan Gu, Yuxin Leng

***Correspondence:** [renliliipb@163.com](mailto:renliliipb@163.com) (Lili Ren), [wangjw28@163.com](mailto:wangjw28@163.com) (Jianwei Wang),

[jiang_chao@zju.edu.cn](mailto:jiang_chao@zju.edu.cn) (Chao Jiang), [lingtonghuang@zju.edu.cn](mailto:lingtonghuang@zju.edu.cn) (Lingtong Huang)

Table of Contents

[Collection of clinical data 4](#_Toc167791881)

[Baseline information 4](#_Toc167791882)

[Time points 4](#_Toc167791883)

[Laboratory tests 4](#_Toc167791884)

[SOFA and APACHE II scores 5](#_Toc167791885)

[Medical imaging 5](#_Toc167791886)

[Drug usage 6](#_Toc167791887)

[Complications 6](#_Toc167791888)

[Clinical Microbiology 7](#_Toc167791889)

[Arterial blood gas results and ventilator parameters 7](#_Toc167791890)

[Follow-up visits 7](#_Toc167791891)

[Outcome assessment for host susceptibility 7](#_Toc167791892)

[Definitions 8](#_Toc167791893)

[Biological sampling procedures 9](#_Toc167791894)

[Collection of BALF, ETA, and sputum 9](#_Toc167791895)

[Negative control of BALF 10](#_Toc167791896)

[Collection of whole blood and serum 10](#_Toc167791897)

[Collection of stool and rectal swabs 10](#_Toc167791898)

[Sample storage 11](#_Toc167791899)

[Sample transport, packaging, and quality assessment 11](#_Toc167791900)

[Methodologies of biological sample processing 12](#_Toc167791901)

[Metagenomic sequencing 12](#_Toc167791902)

[Whole blood RNA-Seq and clinical biomarkers 13](#_Toc167791903)

[Methods of bioinformatics analysis 13](#_Toc167791904)

[Microbial community and function profiling 13](#_Toc167791905)

[Identification of antibiotic resistance genes and virulence genes 13](#_Toc167791906)

[Mobile genetic element identification 14](#_Toc167791907)

[Virome profiling 14](#_Toc167791908)

[Missing data 15](#_Toc167791909)

[Power analysis 15](#_Toc167791910)

[Statistical analysis 15](#_Toc167791911)

[Experimental validation for unknown pathogens 16](#_Toc167791912)

[Pathogen culturing 17](#_Toc167791913)

[In vitro verification using cell lines 17](#_Toc167791914)

[In vivo verification using animal models 17](#_Toc167791915)

[Re-isolation and verification 18](#_Toc167791916)

[Genetic and functional studies 18](#_Toc167791917)

[References 18](#_Toc167791918)

# Collection of clinical data

We will gather a comprehensive set of clinical data from enrolled patients (Figure 2B; Table S2), including baseline information, time points, results of laboratory tests, SOFA and APACHE II scores, medical imaging findings, details of drug usage, recorded complications, clinical microbiology data, arterial blood gas results, and ventilator parameters.

## Baseline information

Baseline information includes the patient's basic information and medical history. Basic information comprises gender, age, height, weight, ethnicity, smoking and drinking history, residence, and birthplace. For medical history, in addition to the diseases covered by the Charlson comorbidity index, the history of transplantation, hypertension, and immunosuppression, as well as specific pre-existing lung diseases and rheumatologic and connective tissue disorders prior to enrollment will be recorded.

## Time points

We will document several specific time points, including the admission date, onset date, hospitalization date, transfer to ICU date, ICU discharge date, hospital discharge date, as well as the start and end times of invasive mechanical ventilation (IMV) and extracorporeal membrane oxygenation (ECMO), both at admission or post-admission (Table S2). For patients with HAP or VAP, the onset date should fall within the hospitalization period. If a patient is re-intubated or undergoes tracheotomy within 12 hours after extubation, this period will be noted as invasive mechanical ventilation time, indicating they have not successfully transitioned from ventilator support.

## Laboratory tests

Laboratory tests for baseline adhere to each hospital’s usual testing procedures, with no compulsory specifications for testing equipment and processing methods. We will collect the following biological indicators:

(1) Immune-inflammatory indicators: hypersensitive C-reactive protein (hsCRP), procalcitonin (PCT), ferritin, complement C4 and C3, immunoglobulins IgM, IgG, and IgA, interleukins IL-2, IL-4, IL-6, IL-8, IL-1β, and IL-10, TNF-α, IFN-γ. In addition to hsCRP, PCT, and ferritin, the tests of other indicators follow the diagnostic and treatment practices of clinicians in each hospital, which is not mandatory. We anticipate some data missing for some hospitals;

(2) Blood coagulation function: D-dimer, activated partial thromboplastin time (APTT), prothrombin time (PT);

(3) Complete blood count: white blood cell count, monocyte count, lymphocyte count, neutrophil count, eosinophil count, basophil count, platelet count, hematocrit (HCT), hemoglobin;

(4) Cardiac biomarkers: B-type natriuretic peptide (BNP), creatine kinase MB (CK-MB), high-sensitivity cardiac troponin I (hs-cTnI);

(5) Renal function: urea nitrogen (UN), creatinine, uric acid (UA);

(6) Liver function: albumin, total bile acid (TBA), total bilirubin, direct bilirubin level (DBiL), alanine aminotransferase (ALT), aspartate aminotransferase (AST), Gamma-glutamyltransferase (GGT), alkaline phosphatase (ALP), lactate dehydrogenase (LDH);

(7) Metabolic biomarkers: triglyceride, cholesterol, high-density lipoprotein (HDL), low-density lipoprotein (LDL), very low-density lipoprotein (VLDL);

(8) Lymphocyte subsets: CD3^+^ T cells, CD3^+^CD4^+^ T cells, CD3^+^CD8^+^ T cells, CD19^+^ B cells, CD3^-^CD16^+^CD56^+^ NK cells. Testing for lymphocyte subsets is not mandatory, and we anticipate some data missing for some hospitals.

## SOFA and APACHE II scores

Given our inclusion of HAP and VAP patients, we will assess SOFA and APACHE II scores within 24 hours of enrollment rather than on the day of ICU transfer. We anticipate that for all patients with CAP and the majority of those with HAP, the time of admission coincides with the time of ICU transfer. The SOFA score will also be evaluated on the fourth day after enrollment, should the patient still be in the ICU at that time. We will report the failure ratio of different organs. Organ failure is considered when the organ-specific SOFA score is two or higher. In China, APACHE II is more commonly utilized than APACHE IV [[1](#_ENREF_1)], leading us to adopt APACHE II scoring in place of APACHE IV scoring.

## Medical imaging

Patients’ medical images and raw data from chest X-rays or CT scans at the time closest to enrollment will be preserved. We anticipate that over 95% of the enrolled patients will undergo chest X-ray or CT scan. In addition to the original imaging data, we will document whether the patient has pleural effusion, pneumothorax, solitary or multiple infectious lesions, pneumonia manifestations in different lung lobes, and the characteristics of infectious lesions, including interstitial changes, consolidation, exudative changes, and the presence of cavities, liquefaction, etc. Lung ultrasound data will not be collected due to the difficulty in unifying the operation of ultrasound across hospitals, which would greatly increase the heterogeneity of the images. All imaging data will be exported every 3 months and saved in portable hard disks.

## Drug usage

To investigate the association of drug use with the microbiome and microbial antibiotic resistance, we will record the time, dosage, and route of drug administration from onset (as per the chief complaint) to 2 weeks after enrollment. Specifically, attention is given to whether patients have received parenteral nutrition, enteral nutrition, probiotics, antibiotics (including via intravenous administration, oral intake, and aerosol inhalation), proton pump inhibitors (PPI), corticosteroids, immunosuppressants, and antiviral drugs.

## Complications

We will monitor patients’ complications during the ICU stay, including acute myocardial injury, and infarction, acute kidney injury, acute respiratory distress syndrome (ARDS), pulmonary embolism (PE), pneumothorax, emerging VAP and HAP, and invasive pulmonary fungal diseases. Definitions of these complications can be found in the following "Definitions" section.

To diagnose invasive pulmonary fungal disease that meets the diagnostic criteria of the Infectious Diseases Society of America (IDSA) [[2](#_ENREF_2),[3](#_ENREF_3)] during the ICU stays, we will conduct G tests and GM tests on different types of biological samples (BALF and blood). If the GM test data for some suspected invasive pulmonary aspergillosis patients are missing, we will conduct supplementary GM tests on the collected biological samples. CT image features of patients suspected of secondary invasive pulmonary aspergillosis will also be documented. If the patient is admitted for invasive pulmonary fungal diseases, the aforementioned clinical data will be collected at enrollment.

## Clinical Microbiology

We will document the infection of bacteria, fungi, and viruses in patients within one month of enrollment, based on the reports from sample cultures, PCR, immunofluorescence, clinical metagenomics, etc. If multi-drug resistant bacteria are present, we will collect relevant information on antimicrobial resistance.

We are interested in several specific pathogen infections, including influenza A, COVID-19, and pneumocystis pneumonia (PCP). For patients with viral infections, we will also gather the CT value of the virus at enrollment, virus typing (if available), antiviral drug usage, and the timing of virus clearance. For patients with PCP, we will focus on whether the patient has received TMP-SMZ prophylaxis and the results of silver hexamine staining. All other clinical data required for PCP are included in the previously mentioned content.

## Arterial blood gas results and ventilator parameters

We will document the patient's initial blood gas results and ventilator parameters at 6-8 am on 3 consecutive days following enrollment. This includes pH, PO_2_, PCO_2_, FiO_2_, lactate levels, ventilator modes (both invasive and noninvasive), peak pressures, positive end-expiratory pressure (PEEP), tidal volume, and respiratory rate.

## Follow-up visits

For patients who survived their hospital stay, we will conduct follow-up assessments at 90 days and 1 year post-enrollment. During these follow-up visits, we will document readmission, EQ-5D-5L scores, and time of death if applicable [[4](#_ENREF_4)]. All ongoing clinical data collection will cease once the patient is discharged from the ICU or in the event of their death.

# Outcome assessment for host susceptibility

Each individual's response to pathogens and response to treatment is unique [[5](#_ENREF_5),[6](#_ENREF_6)]. This is also the core idea of a previous prospective study based on the MARS cohort (ClinicalTrials.gov ID: NCT01905033) [[7](#_ENREF_7)]. The body reacts differently to pathogens, which affects the course of the disease. Some patients with severe pneumonia are considered to have stronger inflammatory responses and some are not, and they have a completely different clinical prognosis. Analyses including latent class analysis (LCA) have been widely used in cohort studies to decode the intricate relationships between clinical and biological features and treatment responses [[8−13](#_ENREF_8)].

The clinical and genetic characteristics of the host are indispensable in our study, as we have previously explored in other diseases [[14](#_ENREF_14),[15](#_ENREF_15)]. We can use the MASS cohort to assess host susceptibility based on a combination of clinical characteristics, including phenotype changes, improvement in surveillance clinical factors, and symptoms. Each component contributes to a comprehensive understanding of the host's symptoms and response to treatment, allowing us to evaluate susceptibility in a multidimensional context.

# Definitions

Definition of severe pneumonia [[16](#_ENREF_16)]: New infection that meets one of the following criteria: the patient receives mechanical ventilation (invasive or non-invasive) due to acute respiratory failure, with a PEEP level of 5 cm or above; The patient receives high flow oxygen therapy with a FiO_2_ of 50% or more and a PaO_2_: FiO_2_ ratio of less than 300; The patient who is treated with an oxygen bag mask, provided that PaO_2_ is lower than the pre-specified indicator.

We report clinical definitions based on existing guidelines and the results of our internal discussions:

(1) The diagnosis of pneumonia follows previous definitions [[17](#_ENREF_17),[18](#_ENREF_18)]. Because all enrolled patients meet the criteria for respiratory failure, diagnosis of infection into two points: (a) Presence of characteristic radiographic changes, and non-infectious causes such as heart failure, trauma, aspiration, and autoimmune pulmonary diseases are ruled out; (b) Conventional microbiological evidence or clinical metagenomics results are consistent with the patient's clinical features. A diagnosis is confirmed if both criteria are met, and suspected if one of the criteria is met. Infection is highly unlikely if neither is met.

(2) Both VAT and VAP are considered ventilator-associated respiratory infections (VARI). In this study, we focus on VAP [[19](#_ENREF_19),[20](#_ENREF_20)]. The diagnosis of VAP includes: 1. Invasive mechanical ventilation for more than 48 hours; 2. New or progressive infiltrates on lung CT or X-ray, combined with two or more of the following symptoms: fever, increased neutrophil count (> 10×10^9/L) or decreased neutrophil count (< 5×10^9/L), and purulent sputum.

(3) The diagnosis of HAP includes [[21](#_ENREF_21),[22](#_ENREF_22)] : Symptom onset at least 48 hours post-hospital admission, with invasive mechanical ventilation for less than 48 hours; New or progressive infiltrates on lung CT or X-ray, combined with two or more of the following symptoms: fever, increased neutrophil count (> 10×10^9^/L) or decreased neutrophil count (< 5×10^9^/L), and purulent sputum.

(4) Charlson comorbidity index refers to the previous definition [[23](#_ENREF_23)].

(5) The definition of acute kidney injury refers to the KDIGO guidelines [[24](#_ENREF_24),[25](#_ENREF_25)]. Stage 1: Serum creatinine criteria include an absolute increase ≥ 0.3 mg/dL (> 26.5 μmol/L) or a relative increase ≥ 50% from baseline, but < 1 times the baseline; or urine output < 0.5 mL/(kg·h) (for more than 6 hours but less than 12 hours); Stage 2: Serum creatinine criteria include a relative increase ≥ 1 time but < 2 times from baseline; or urine output < 0.5 mL/(kg·h) (for more than 12 hours but less than 24 hours); Stage 3: Serum creatinine criteria include an increase to > 4.0 mg/dL (> 353.6 μmol/L) or a relative increase ≥ 2 times from baseline; or the initiation of continuous renal replacement therapy (CRRT); or urine output < 0.3 mL/(kg·h) (for more than 24 hours) or anuria for ≥ 12 hours.

(6) The definition of ARDS refers to the latest ATS guidelines [[26](#_ENREF_26),[27](#_ENREF_27)]. Use of high-flow nasal oxygen (HFNO) at a minimum flow rate of ≥ 30 L/min, or non-invasive ventilation (NIV) or continuous positive airway pressure (CPAP) with positive end-expiratory pressure (PEEP) of at least 5 cm H_2_O. Low oxygenation is identified by a PaO_2_/FiO_2_ ratio ≤ 300 mmHg or an SpO_2_/FiO_2_ ratio ≤ 315 mmHg with SpO_2_ ≤ 97%. Bilateral opacities are retained as imaging criteria, which may be assessed through chest X-ray, CT, or ultrasound (if the operator is well-trained).

(7) The definition of acute myocardial injury or myocardial infarction is the same as previously described [[28](#_ENREF_28),[29](#_ENREF_29)]. The definition of acute myocardial injury is an elevation in cTnI levels greater than the upper reference limit, without accompanying acute myocardial ischemic changes. The definition of myocardial infarction is an elevation in cTnI levels greater than the upper reference limit, accompanied by acute myocardial ischemic changes.

(8) The diagnosis of pulmonary embolism relies on clinical judgment. Due to limitations in ICU conditions, CT pulmonary angiography is not mandatory. However, researchers need to document the diagnostic criteria [[30](#_ENREF_30)].

(9) The diagnosis of primary or secondary invasive pulmonary fungal is the same as the diagnostic criteria outlined by the Infectious Diseases Society of America (IDSA) [[3](#_ENREF_3)].

# Biological sampling procedures

## Collection of BALF, ETA, and sputum

In all participating medical centers, bronchoalveolar lavage (BAL) is the standard procedure for diagnosing severe pneumonia in intubated patients without contraindications to BAL.

For intubated patients, the diseased lung segment is identified based on the CT scan or X-ray, and BALF and ETA are obtained using a fiberoptic bronchoscope. Firstly, secretions visible in the primary bronchi are aspirated using a fiberoptic bronchoscope to obtain ETA. The bronchoscope is then cleaned twice with normal saline before commencing BAL. To mitigate potential BAL complications, and consider the use of mini-BAL (20 ml saline lavage with blind insertion) in lung microbiome studies [[31](#_ENREF_31)], we use a 50 ml saline lavage volume instead of the previously reported 120 ml saline volume in some studies [[32](#_ENREF_32),[33](#_ENREF_33)]. The bronchoscope is inserted into the affected lung segment, and a total of 50 ml saline is injected in two separate doses, followed by aspiration 5 seconds later. Recovered fluid exceeding 30% of the lavage volume, i.e., greater than 15 ml, is considered eligible. Within 5 minutes after aspiration, the recovered fluid is divided into two tubes in a clean area, with an equal volume of 2X DNA/RNA Shield (Catalog: R1200-125, ZYMO RESEARCH) added to one tube and thoroughly mixed.

For non-intubated patients, the sputum or bronchial aspirate is collected through a suction tube. All specimens are then stored in a -80°C freezer.

## Negative control of BALF

Every time a new batch of BALF sampling consumables is acquired, each medical center collects two negative controls. This involves injecting 20 ml of the new batch of saline into the new fiberoptic bronchoscope and then into the new batch of BALF sampling tubes. Subsequently, similar to the processing of the BALF samples, the liquid in the BALF sampling tubes is dispensed into two tubes, with an equal volume of 2X DNA/RNA Shield added to one of the tubes.

## Collection of whole blood and serum

The serum is collected and retained in 6 ml aliquots at each time point. Whole blood is collected in a volume of 3 ml and then transferred into a 15 ml centrifuge tube. To preserve the sample, an equal volume of 2X DNA/RNA Shield (Catalog: R1200-125, ZYMO RESEARCH) is added. After thorough mixing, the sample is stored in a -80°C freezer.

## Collection of stool and rectal swabs

We collect either feces or rectal swabs with visible fecal components, with fecal samples being the preferred specimen. Fecal samples are obtained using a standardized approach. In cases where no stool is passed, rectal swabs are collected. The procedure for collecting rectal swabs includes the following steps: 1) Remove the flocked swab from its packaging, holding it by the end of the plastic handle to avoid contact between the cotton tip and any surfaces. 2) Insert the swab directly into the rectum, approximately 2-3 cm into the anal canal, rotating the swab three times, then withdraw. The presence of feces on the swab indicates correct sampling. 3) Break the swab at the neck and place it into a freezer tube. Both fecal samples and rectal swabs are promptly stored at -80°C without the use of any cryopreservative.

## Sample storage

Each hospital will seal the samples with Parafilm, store them in a specialized -80°C refrigerator, and attach customized labels. All the refrigerators and centrifuges in the study were purchased and supplied to each medical center from the same source.

## Sample transport, packaging, and quality assessment

All non-infectious biological specimens are transferred to the National Institute of Pathogen Biology, Chinese Academy of Medical Sciences & Peking Union Medical College (CAMS & PUMC) via dry ice every 2 months and aliquoted by experienced technicians in a biosafety cabinet. Negative controls are also established. This involves pipetting phosphate-buffered saline (PBS) using each batch of pipette tips and dispensing it into EP tubes of the same batch. Subsequently, all specimens will be transferred to the First Affiliated Hospital of Zhejiang University School of Medicine for management and preservation.

The quality of bronchoalveolar lavage fluid (BALF) samples is rigorously assessed during the aliquoting phase by pathologists. A BALF containing more than 5% of bronchial (squamous or ciliated epithelial) cells or less than 50,000 cells/ml was said of “poor quality”. Otherwise, the BAL fluid was considered of “good quality” [[34](#_ENREF_34)]. Furthermore, we also standardized the lavage volume at 50 mL based on Kamel et al.’s findings that sample quality increases with volume up to 50 mL, beyond which the improvement plateaus. Using more than 50 mL could lead to additional complications, hence our choice of this volume to maximize quality without increasing risk.

# Methodologies of biological sample processing

We plan to use the following procedures for DNA extraction, library construction, and metagenomic sequencing for microbiomes from respiratory samples, fecal samples, and rectal swab samples. Given the low microbial DNA concentration in respiratory samples, enhanced methods for host DNA depletion and DNA extraction will be utilized. Moreover, we plan to conduct human blood RNA-seq to investigate the host transcriptome and virome.

## Metagenomic sequencing

Respiratory samples, such as BALF and sputum, will undergo initial treatment with sputasol, followed by incubation to liquefy the samples. Sputasol-treated samples are then subjected to the procedure for host DNA depletion, which is modified from a previously reported saponin-based differential lysis method [[35](#_ENREF_35),[36](#_ENREF_36)]. Post-depletion, samples are sonicated to lyse microbial cells to extract the DNA. Protein precipitation is achieved with ammonium acetate, followed by DNA concentration using sodium acetate and ethanol precipitation. After the final centrifugation and washing steps, the DNA pellet is dried and resuspended in nuclease-free water. DNA from crude fecal and rectal swab samples will be extracted using the DNeasy PowerLyzer PowerSoil Kit (Qiagen, Hilden, Germany) following the manufacturer’s instructions. DNA will be quantified using a Qubit Fluorometer (ThermoFisher, UK). Samples with DNA concentration > 0.1 ng/µl after host DNA removal will be sequenced.

Metagenomic libraries with an insert size of 350 bp will be prepared using a VAHTS Universal Plus DNA Library Prep Kit (Vazyme #ND617, Nanjing, China) according to the manufacturer's protocol. The quantity and quality of libraries will be assessed using the Qubit dsDNA HS Assay Kit (Vazyme #EQ121, Nanjing, China) and Qsep100 (BiOptic, New Taipei City, China). The final libraries will be sequenced for 2×150bp paired-end sequencing on the Illumina NovaSeq 6000 platform or equivalent instruments. We aim to sequence 10-30 Gb per sample depending on the cost of sequencing and ensuring comprehensive coverage for detecting a wide range of microbial taxa.

The raw sequencing reads will be processed with fastp [[37](#_ENREF_37)] to remove adaptor sequences and trim low-quality bases. Human reads will be removed using a high-performance two-stage bioinformatics approach [[38](#_ENREF_38)], in which reads will be first aligned to human genome GRCh38 using Bowtie2 [[39](#_ENREF_39)] and undergo a secondary alignment using HISAT2 [[40](#_ENREF_40)].

## Whole blood RNA-Seq and clinical biomarkers

Trizol reagent (Invitrogen, USA) will be used to extract whole blood RNA [22]. RNA quality will be determined with the NanoDrop One (Thermo Fisher Scientific, USA) [[41](#_ENREF_41)]. Libraries will be constructed starting with 500 ng of total RNA using the NEBNext Ultra II Directional RNA Library Prep Kit (Illumina, USA). Sequencing will be performed on the NovaSeq 6000 platform (Illumina, USA) with paired-end 2 × 150 bp reads.

Clinical biomarkers such as cell types and counts, blood glucose, blood lipids, and markers for liver, kidney, gallbladder, and thyroid function will be measured using automated equipment in the hospital laboratory.

# Methods of bioinformatics analysis

## Microbial community and function profiling

For taxonomic profiling and relative abundance estimating, we plan to use Kraken2 based on a comprehensive custom index constructed with all genomes of bacteria, fungi, archaea, and protozoa from RefSeq and GenBank. Functional profiling will be performed using HUMAnN3, with the abundance of functional pathways classified against UniRef90 and ChocoPhlAn databases.

We will assemble metagenomic data from both respiratory and fecal/rectal samples. De novo genome assembly and draft genome construction will be performed with the pipeline of MetaWRAP [[42](#_ENREF_42)]. In this pipeline, contigs with < 1000 bp will be discarded in assemblies. Quality assessment of MAGs will be based on completeness and contamination evaluated with CheckM. Only MAGs with ≥ 50% completeness and < 10% contamination will be used for subsequent analyses. To remove the redundancy of MAGs, we will cluster them using dRep with 99% nucleotide identity (ANI). The retaining bins will further be clustered into species-level representative genomes (SRGs) at the threshold of 95% ANI. The phylogenetic inference of MAGs will be conducted by GTDB-Tk based on the Genome Taxonomy Database (GTDB; https://gtdb.ecogenomic.org/) [[43](#_ENREF_43),[44](#_ENREF_44)]. Maximum-likelihood trees will be built by IQ-TREE with the protein sequence alignments produced by GTDB-Tk. Trees will be visualized and annotated with ggtreeExtra [[45](#_ENREF_45)].

## Identification of antibiotic resistance genes and virulence genes

For the read-based identification of ARGs, all processed reads will be classified and annotated against the SARG database using ARG-OAP with default parameters. ARGs will be categorized into ARG types (the class of antibiotics targeted by the gene) and subtypes (functional gene annotation). ARG abundance will be normalized by the number of 16S reads, and richness was calculated as the total number of ARGs. For the contig-based profiling of ARGs in metagenomic assemblies, contigs will be annotated against CARD using Resistance Gene Identifier (RGI), a command-line tool downloaded from the CARD website (https://card.mcmaster.ca/). Genes will be identified using Prodigal [[46](#_ENREF_46)] with ‘meta’ mode and annotated using KofamKOALA [[47](#_ENREF_47)] to search against the Kyoto Encyclopedia of Genes and Genomes (KEGG) [[48](#_ENREF_48)]. Virulence genes will be screened against VFDB [[49](#_ENREF_49)] using ABRicate (https://github.com/tseemann/abricate).

## Mobile genetic element identification

All the assembled contigs will be analyzed to explore the full spectrum of MGEs using MobileElementFinder [[50](#_ENREF_50)], which categorizes MGEs into Miniature Inverted Repeats (MITEs), Insertion Sequences (ISs), Composite Transposons (ComTns), Unit Transposons (Tns), Integrative Conjugative Elements (ICEs), Integrative Mobilizable Elements (IMEs), and Cis-Mobilizable Elements (CIMEs). Species-level characterization of MGEs will be performed for high-quality MAGs. Only high-quality MGEs will be retained if the identity > 90% and coverage > 95%. An antibiotic resistance gene is considered associated with MGEs if it is located within an MGE or with an interval < 5 kbp [[51](#_ENREF_51)]. Additionally, we plan to apply some tools for Horizontal gene transfer (HGT) detection based on metagenomics, such as Daisy [[52](#_ENREF_52)] and MetaCHIP [[53](#_ENREF_53)].

## Virome profiling

For bronchoalveolar lavage fluid (BALF) and fecal samples, we will employ VirSorter2, CheckV, and DRAMv software to detect DNA and RNA viruses from metagenomic and metatranscriptomic sequencing data [[54](#_ENREF_54)].

Sequencing data from whole transcriptome libraries of blood samples will be analyzed for the presence of RNA sequences corresponding to known human viral pathogens using the sequence-based ultra-rapid pathogen identification (SURPI) computational pipeline [[55](#_ENREF_55)]. After computationally subtracting human reads, the remaining reads will be aligned against all microbial sequences in the NCBI GenBank database. The SNAP aligner [[56](#_ENREF_56)] will be used at moderate stringency (edit distance = 12) to align reads to the NCBI nucleotide nt database, allowing for the detection of reads with ≥ 90% nucleotide identity to known viruses, while the RAPSearch [[57](#_ENREF_57)] will be used to detect divergent reads from potential novel viruses by translated nucleotide alignment to the NCBI protein nr database. A rapid taxonomic classification algorithm based on the lowest common ancestor will be incorporated into SURPI, as previously described [[58](#_ENREF_58)].

## Missing data

If an entire dataset from a specific medical center is missing, we will exclude that center in analyses requiring the missing dataset. For individual missing data points, we will employ multiple imputation techniques [[59](#_ENREF_59),[60](#_ENREF_60)].

## Power analysis

We performed power analysis to calculate the sufficient sample size for statistics among CAP, HAP, and VAP, using the pwr.anova.test function from the R package *pwr*. Assuming a medium effect size (f = 0.25) and 80% power in one-way ANOVA, the minimum number of participants in each group was estimated to be 53. Of the 286 patients enrolled, 194 (67.83%) had CAP, 69 (24.13%) had HAP, and 15 (5.24%) had VAP. Accordingly, among the 2000 patients recruited in the future, it is expected that the number of CAP, HAP, and VAP patients can reach 1357, 483, and 105, which is much larger than the required sample size of 53. Therefore, the 2000 patients we plan to enroll are sufficient to meet statistical needs.

We performed power analysis to calculate the sufficient sample size for statistics on longitudinal data with 3 time points, using the pwr.f2.test function from the R package *pwr*. Assuming a medium effect size (f = 0.25), 80% power, and 3 numerators (i.e., 3 time points) in a general linear model, the minimum number of participants was estimated to be 47.7. Therefore, among the 2000 patients, the 500 patients with ≥ 3 time points we estimated to include should provide sufficient power to explore the dynamic changes in the lung microbiome.

## Statistical analysis

Microbiome and resistome alpha diversity metrics will be analyzed using the *phyloseq* and *vegan* packages. We will apply the vegdist() function from the *vegan* R package (v2.6-4) to calculate the Bray-Curtis dissimilarity. Subsequently, classical multidimensional scaling will be carried out to obtain different principal coordinates. We will perform the permutational multivariate analysis of variance (PERMANOVA) by the vegan::adonis2() function. Kruskal–Wallis test with Dunn’s post-hoc test will be used for multiple comparisons. Correlations will be assessed using the ‘cor.test’ function of the *stats* package. Kolmogorov–Smirnov test will be used for the MGE length distribution comparisons. The R packages *survival* and *survminer* will be used to perform survival analysis. The R package *ReporterScore* will be applied to perform generalized reporter score-based enrichment analysis for omics data [[61](#_ENREF_61)].

We will employ linear mixed effects (LME) models to analyze the relationship between microbiome profiles and clinical data (including clinical outcomes). This approach is particularly suited to our study due to its ability to handle data complexities such as repeated measures from the same subjects and subjects nested within centers.

The LME modeling includes: (1) Dependent variables: Clinical outcomes, such as treatment response and progression-free survival; (2) Independent variables: Microbiome diversity metrics and abundance of specific microbial taxa and genes; (3) Fixed effects: Key clinical covariates, including age, gender, baseline health status, and other relevant factors; (4) Random effects: Intra-subject and inter-center variability to account for longitudinal measures and center-specific differences.

To account for potential confounders, we will: (1) Adjust for known confounders: Include demographic factors, lifestyle variables (e.g., drinking and smoking status), and clinical characteristics (e.g., comorbidities) as fixed effects in the LME models; (2) Conduct sensitivity analyses: Test different model specifications and the inclusion/exclusion of certain covariates to ensure robustness; (3) Use propensity score matching: Balance the distribution of confounders between comparison groups, reducing bias.

We will also validate our findings through N-fold cross-validations within the ZJ cohort and, where possible, use the non-ZJ cohort for external validation to ensure the generalizability of our results.

# Experimental validation for unknown pathogens

To verify the pathogenicity of a newly discovered pneumonia-causing pathogen, whether bacterial, viral, or fungal, identified through omics studies, a combination of in vitro and in vivo experiments is essential. We will identify potential findings from our omics analysis that require verification in the future and plan our experiments accordingly. Below we describe the potential experimental procedures.

### Pathogen culturing

Cultivate the pathogen using appropriate growth media, for example, luria broth (LB) agar for bacteria [[62](#_ENREF_62)], specific cell culture lines for viruses [[63](#_ENREF_63)], Sabouraud agar for fungi [[64](#_ENREF_64)]. Three-dimensional air-liquid interface culture system may used to facilitate the cultivation process and ensure stable [[65](#_ENREF_65)].

### In vitro verification using cell lines

Relevant cell lines: (1) A549 cells: Human lung carcinoma cells commonly used for respiratory pathogen studies (e.g., bacteria, viruses) [[66](#_ENREF_66),[67](#_ENREF_67)]; (2) Vero E6 cells: Kidney cells of an African green monkey, commonly used to isolate, propagate, and study respiratory viruses like MERS-CoV and SARS-CoV [[63](#_ENREF_63)]; (3) CuFi-8 cells: Immortalized human airway epithelial cell line derived from airway epithelial cells of a donor cystic fibrosis patient, which were used in study for fungi and viruses [[68](#_ENREF_68)]; (4) NHBE cells: Normal human bronchial epithelial cells for a more physiologically relevant model [[69](#_ENREF_69)].

Assays for pathogenicity: (1) Cytotoxicity assays: Evaluate cell viability post-infection using LDH or MTT assays to determine cell damage and death caused by the pathogen [[70](#_ENREF_70),[71](#_ENREF_71)]; (2) Cytokine production: Measure the release of cytokines such as IL-6, IL-8, and TNF-alpha using ELISA to assess the inflammatory response [[72](#_ENREF_72)].

### In vivo verification using animal models

Mouse models that are commonly used due to genetic similarity to humans and available genetic tools, including: (1) BALB/c mice: Frequently used for respiratory infection studies [[73](#_ENREF_73)]; (2) C57BL/6 mice: Used for immune response studies [[74](#_ENREF_74)]; (3) Transgenic Mice: Expressing human receptors for specific pathogens, such as hACE2 mice for SARS-CoV-2 [[75](#_ENREF_75)].

Experimental procedures: (1) Infection routes: Administer the pathogen via intranasal instillation or intratracheal injection to mimic natural infection [[76](#_ENREF_76),[77](#_ENREF_77)]; (2) Symptom monitoring: Observe animals for clinical signs of pneumonia, including weight loss, respiratory distress, and lethargy; (3) Pathological assessment: Perform necropsy and histopathological examination of lung tissues to assess inflammation, tissue damage, and pathogen spread.

### Re-isolation and verification

(1) Re-isolation of pathogen: Isolate the pathogen from infected tissues to confirm it causes the observed symptoms, following Koch's postulates [[78](#_ENREF_78)]; (2) Molecular verification: Use PCR or sequencing to verify the identity of the pathogen re-isolated from the animal models.

### Genetic and functional studies

(1) Mutagenesis studies: For bacteria and fungi, create gene knockouts to identify virulence factors by comparing the pathogenicity of wild-type and mutant strains; (2) Transcriptomic and proteomic analyses: Analyze host-pathogen interactions and host immune response during infection to identify key pathogenic mechanisms and potential therapeutic targets.

# REFERENCES

1. Li, Andrew, Lowell Ling, Hanyu Qin, Yaseen M. Arabi, Sheila Nainan Myatra, Moritoki Egi, Je Hyeong Kim, et al. 2022. “Epidemiology, Management, and Outcomes of Sepsis in ICUs among Countries of Differing National Wealth across Asia.” *American Journal of Respiratory and Critical Care Medicine* 206: 1107-1116. https://doi.org/10.1164/rccm.202112-2743OC

2. De Pauw, Ben, Thomas J. Walsh, J. Peter Donnelly, David A. Stevens, John E. Edwards, Thierry Calandra, Peter G. Pappas, et al. 2008. “Revised definitions of invasive fungal disease from the European Organization for Research and Treatment of Cancer/Invasive Fungal Infections Cooperative Group and the National Institute of Allergy and Infectious Diseases Mycoses Study Group (EORTC/MSG) Consensus Group.” *Clinical Infectious Diseases* 46: 1813-1821. https://doi.org/10.1086/588660

3. Bassetti, Matteo, Elie Azoulay, Bart-Jan Kullberg, Markus Ruhnke, Shmuel Shoham, Jose Vazquez, Daniele Roberto Giacobbe, et al. 2021. “EORTC/MSGERC Definitions of Invasive Fungal Diseases: Summary of Activities of the Intensive Care Unit Working Group.” *Clinical Infectious Diseases* 72: S121-s127. https://doi.org/10.1093/cid/ciaa1751

4. Pisani, Margaret A., So Yeon Joyce Kong, Stanislav V. Kasl, Terrence E. Murphy, Katy L. B. Araujo, and Peter H. Van Ness. 2009. “Days of delirium are associated with 1-year mortality in an older intensive care unit population.” *American Journal of Respiratory and Critical Care* 180: 1092-1097. https://doi.org/10.1164/rccm.200904-0537OC

5. Bourdon, Marie, Caroline Manet, Xavier Montagutelli. 2020. “Host genetic susceptibility to viral infections: the role of type I interferon induction.” *Genes & Immunity* 21: 365-379. https://doi.org/10.1038/s41435-020-00116-2

6. Casadevall, Arturo, Liise-anne Pirofski. 2018. “What Is a Host? Attributes of Individual Susceptibility.” *Infection and Immunity* 86: 10.1128/iai.00636-00617. https://doi.org/doi:10.1128/iai.00636-17

7. Peters-Sengers, Hessel, Joe M. Butler, Fabrice Uhel, Marcus J. Schultz, Marc J. Bonten, Olaf L. Cremer, Brendon P. Scicluna, et al. 2022. “Source-specific host response and outcomes in critically ill patients with sepsis: a prospective cohort study.” *Intensive Care Medicine* 48: 92-102. https://doi.org/10.1007/s00134-021-06574-0

8. Maddali, Manoj V., Matthew Churpek, Tai Pham, Emanuele Rezoagli, Hanjing Zhuo, Wendi Zhao, June He, et al. 2022. “Validation and utility of ARDS subphenotypes identified by machine-learning models using clinical data: an observational, multicohort, retrospective analysis.” *The Lancet Respiratory Medicine* 10: 367-377. https://doi.org/10.1016/S2213-2600(21)00461-6

9. Sinha, Pratik, V. Eric Kerchberger, Andrew Willmore, Julia Chambers, Hanjing Zhuo, Jason Abbott, Chayse Jones, et al. 2023. “Identifying molecular phenotypes in sepsis: an analysis of two prospective observational cohorts and secondary analysis of two randomised controlled trials.” *The Lancet Respiratory Medicine* 11: 965-974. https://doi.org/10.1016/S2213-2600(23)00237-0

10. Sinha, Pratik, Kevin L. Delucchi, Daniel F. McAuley, Cecilia M. O'Kane, Michael A. Matthay, Carolyn S. Calfee. 2020. “Development and validation of parsimonious algorithms to classify acute respiratory distress syndrome phenotypes: a secondary analysis of randomised controlled trials.” *The Lancet Respiratory Medicine* 8: 247-257. https://doi.org/10.1016/S2213-2600(19)30369-8

11. Redaelli, Simone, Dario von Wedel, Maxime Fosset, Aiman Suleiman, Guanqing Chen, Julie Alingrin, Michelle N. Gong, et al. 2023. “Inflammatory subphenotypes in patients at risk of ARDS: evidence from the LIPS-A trial.” *Intensive Care Medicine* 49: 1499-1507. https://doi.org/10.1007/s00134-023-07244-z

12. Sinha, Pratik, Kevin L. Delucchi, B. Taylor Thompson, Daniel F. McAuley, Michael A. Matthay, Carolyn S. Calfee, Nhlbi Ards Network for the. 2018. “Latent class analysis of ARDS subphenotypes: a secondary analysis of the statins for acutely injured lungs from sepsis (SAILS) study.” *Intensive Care Medicine* 44: 1859-1869. https://doi.org/10.1007/s00134-018-5378-3

13. Heijnen, Nanon F. L., Laura A. Hagens, Marry R. Smit, Olaf L. Cremer, David S. Y. Ong, Tom van der Poll, Lonneke A. van Vught, et al. 2021. “Biological Subphenotypes of Acute Respiratory Distress Syndrome Show Prognostic Enrichment in Mechanically Ventilated Patients without Acute Respiratory Distress Syndrome.” *American Journal of Respiratory and Critical Care Medicine* 203: 1503-1511. https://doi.org/10.1164/rccm.202006-2522OC

14. Huang, Lingtong, Xia Zheng, Xiaohan Huang, Lijun Wang, Xueling Fang, Guojun He, Meng Tang, Huixian Shi, Hongliu Cai. 2023. “Alveolar Hemorrhage in Idiopathic Multicentric Castleman’s Disease.” *American Journal of Respiratory and Critical Care Medicine* 208: 613-615. https://doi.org/10.1164/rccm.202204-0780IM

15. Gu, Yudan, Shafei Liu, Xiaohan Huang, Lingtong Huang, Lingling Tang. 2023. “Polychondritis in a child.” *The Lancet Rheumatology* 5: e695-e696. https://doi.org/10.1016/S2665-9913(23)00186-8

16. Mandell, Lionel A., Richard G. Wunderink, Antonio Anzueto, John G. Bartlett, G. Douglas Campbell, Nathan C. Dean, Scott F. Dowell, et al. 2007. “Infectious Diseases Society of America/American Thoracic Society Consensus Guidelines on the Management of Community-Acquired Pneumonia in Adults.” *Clinical Infectious Diseases* 44: S27-S72. https://doi.org/10.1086/511159

17. Calandra, Thierry and Jonathan Cohen. 2005. “The international sepsis forum consensus conference on definitions of infection in the intensive care unit.” *Critical Care Medicine* 33: 1538-1548. https://doi.org/10.1097/01.ccm.0000168253.91200.83

18. Horan, Teresa C., Mary Andrus, and Margaret A. Dudeck. 2008. “CDC/NHSN surveillance definition of health care-associated infection and criteria for specific types of infections in the acute care setting.” *American Journal of Infection Control* 36: 309-332. https://doi.org/10.1016/j.ajic.2008.03.002

19. Torres, Antoni, Michael S. Niederman, Jean Chastre, Santiago Ewig, Patricia Fernandez-Vandellos, Hakan Hanberger, Marin Kollef, et al. 2017. “International ERS/ESICM/ESCMID/ALAT guidelines for the management of hospital-acquired pneumonia and ventilator-associated pneumonia.” *European Respiratory Journal* 50: 1700582. https://doi.org/10.1183/13993003.00582-2017

20. Martin-Loeches, Ignacio, Pedro Povoa, and Saad Nseir. 2023. “Ventilator associated tracheobronchitis and pneumonia: one infection with two faces.” *Intensive Care Medicine* 49: 996-999. https://doi.org/10.1007/s00134-023-07086-9

21. Kalil, Andre C., Mark L. Metersky, Michael Klompas, John Muscedere, Daniel A. Sweeney, Lucy B. Palmer, Lena M. Napolitano, et al. 2016. “Management of Adults With Hospital-acquired and Ventilator-associated Pneumonia: 2016 Clinical Practice Guidelines by the Infectious Diseases Society of America and the American Thoracic Society.” *Clinical Infectious Diseases* 63: e61-e111. https://doi.org/10.1093/cid/ciw353

22. 2005. “Guidelines for the management of adults with hospital-acquired, ventilator-associated, and healthcare-associated pneumonia.” *American Journal of Respiratory and Critical Care Medicine* 171: 388-416. https://doi.org/10.1164/rccm.200405-644ST

23. Charlson, Mary E., Peter Pompei, Kathy L. Ales, and C. Ronald MacKenzie. 1987. “A new method of classifying prognostic comorbidity in longitudinal studies: Development and validation.” *Journal of Chronic Diseases* 40: 373-383. https://doi.org/10.1016/0021-9681(87)90171-8

24. Khwaja, Arif. 2012. “KDIGO Clinical Practice Guidelines for Acute Kidney Injury.” *Nephron Clinical Practice* 120: c179-c184. https://doi.org/10.1159/000339789

25. Zarbock, Alexander, Mitra K. Nadim, Peter Pickkers, Hernando Gomez, Samira Bell, Michael Joannidis, Kianoush Kashani, et al. 2023. “Sepsis-associated acute kidney injury: consensus report of the 28th Acute Disease Quality Initiative workgroup.” *Nature Reviews Nephrology* 19: 401-417. https://doi.org/10.1038/s41581-023-00683-3

26. Qadir, Nida, Sarina Sahetya, Laveena Munshi, Charlotte Summers, Darryl Abrams, Jeremy Beitler, Giacomo Bellani, et al. 2024. “An Update on Management of Adult Patients with Acute Respiratory Distress Syndrome: An Official American Thoracic Society Clinical Practice Guideline.” *American Journal of Respiratory and Critical Care Medicine* 209: 24-36. https://doi.org/10.1164/rccm.202311-2011ST

27. Grasselli, Giacomo, Carolyn S. Calfee, Luigi Camporota, Daniele Poole, Marcelo B. P. Amato, Massimo Antonelli, Yaseen M. Arabi, et al. 2023. “ESICM guidelines on acute respiratory distress syndrome: definition, phenotyping and respiratory support strategies.” *Intensive Care Medicine* 49: 727-759. https://doi.org/10.1007/s00134-023-07050-7

28. Byrne, Robert A, Xavier Rossello, J J Coughlan, Emanuele Barbato, Colin Berry, Alaide Chieffo, Marc J Claeys, et al. 2023. “2023 ESC Guidelines for the management of acute coronary syndromes: Developed by the task force on the management of acute coronary syndromes of the European Society of Cardiology (ESC).” *European Heart Journal* 44: 3720-3826. https://doi.org/10.1093/eurheartj/ehad191

29. Thygesen, Kristian, Joseph S. Alpert, Allan S. Jaffe, Bernard R. Chaitman, Jeroen J. Bax, David A. Morrow, Harvey D. White. 2018. “Fourth Universal Definition of Myocardial Infarction (2018).” *Journal of the American College of Cardiology* 72: 2231-2264. https://doi.org/10.1016/j.jacc.2018.08.1038

30. Konstantinides, Stavros V., Guy Meyer, Cecilia Becattini, Héctor Bueno, Geert-Jan Geersing, Veli-Pekka Harjola, Menno V. Huisman, et al. 2019. “2019 ESC Guidelines for the diagnosis and management of acute pulmonary embolism developed in collaboration with the European Respiratory Society (ERS).” *European Respiratory Journal* 54: 1901647. https://doi.org/10.1183/13993003.01647-2019

31. Erden, Veysel, Gökcen Basaranoglu, Ismet Beycan, Hamdi Delatioğlu, Nıhal Sanlı Hamzaoglu. 2003. “Reproducibility of mini-BAL culture results using 10 ml or 20 ml instilled fluid.” *Intensive Care Medicine* 29: 1856-1856. https://doi.org/10.1007/s00134-003-1964-z

32. Martin-Loeches, Ignacio, Jean Chastre, Richard G. Wunderink. 2023. “Bronchoscopy for diagnosis of ventilator-associated pneumonia.” *Intensive Care Medicine* 49: 79-82. https://doi.org/10.1007/s00134-022-06898-5

33. Dickson, Robert P., Marcus J. Schultz, Tom van der Poll, Laura R. Schouten, Nicole R. Falkowski, Jenna E. Luth, Michael W. Sjoding, et al. 2020. “Lung Microbiota Predict Clinical Outcomes in Critically Ill Patients.” *American Journal of Respiratory and Critical Care Medicine* 201: 555-563. https://doi.org/10.1164/rccm.201907-1487OC

34. Kamel, Toufik, Julie Helms, Ralf Janssen-Langenstein, Achille Kouatchet, Antoine Guillon, Jeremy Bourenne, Damien Contou, et al. 2020. “Benefit-to-risk balance of bronchoalveolar lavage in the critically ill. A prospective, multicenter cohort study.” *Intensive Care Medicine* 46: 463-474. https://doi.org/10.1007/s00134-019-05896-4

35. Charalampous, Themoula, Gemma L. Kay, Hollian Richardson, Alp Aydin, Rossella Baldan, Christopher Jeanes, Duncan Rae, et al. 2019. “Nanopore metagenomics enables rapid clinical diagnosis of bacterial lower respiratory infection.” *Nature Biotechnology* 37: 783-792. https://doi.org/10.1038/s41587-019-0156-5

36. Minghui Cheng, Yingjie Xu, Xiao Cui, Xin Wei, Yundi Chang, Jun Xu, Cheng Lei, Lei Xue, Zhang Wang, Lingtong Huang, Min Zheng, Hong Luo, Yuxin Leng, Chao Jiang. 2023. “Deep longitudinal lower respiratory tract microbiome profiling reveals genome-resolved functional and evolutionary dynamics in critical illness.” *Research Square* PREPRINT (Version 1): https://doi.org/10.21203/rs.3.rs-3494655/v1

37. Chen, Shifu, Yanqing Zhou, Yaru Chen, Jia Gu. 2018. “fastp: an ultra-fast all-in-one FASTQ preprocessor.” *Bioinformatics* 34: i884-i890. https://doi.org/10.1093/bioinformatics/bty560

38. Bush, Stephen J., Thomas R. Connor, Tim E.A. Peto, Derrick W. Crook, A. Sarah Walker. 2020. “Evaluation of methods for detecting human reads in microbial sequencing datasets.” *Microbial Genomics* 6: https://doi.org/10.1099/mgen.0.000393

39. Langmead, Ben, Steven L. Salzberg. 2012. “Fast gapped-read alignment with Bowtie 2.” *Nature Methods* 9: 357-359. https://doi.org/10.1038/nmeth.1923

40. Kim, Daehwan, Joseph M. Paggi, Chanhee Park, Christopher Bennett, Steven L. Salzberg. 2019. “Graph-based genome alignment and genotyping with HISAT2 and HISAT-genotype.” *Nature Biotechnology* 37: 907-915. https://doi.org/10.1038/s41587-019-0201-4

41. Chen, Si, Zhan Li, Haolong Li, Xiaoli Zeng, Hui Yuan, Yongzhe Li. 2023. “RNA Sequencing of Whole Blood in Premature Coronary Artery Disease: Identification of Novel Biomarkers and Involvement of T Cell Imbalance.” *Journal of Cardiovascular Translational Research* https://doi.org/10.1007/s12265-023-10465-8

42. Uritskiy, Gherman V., Jocelyne DiRuggiero, James Taylor. 2018. “MetaWRAP—a flexible pipeline for genome-resolved metagenomic data analysis.” *Microbiome* 6: 158. https://doi.org/10.1186/s40168-018-0541-1

43. Chaumeil, Pierre-Alain, Aaron J Mussig, Philip Hugenholtz, Donovan H Parks. 2022. “GTDB-Tk v2: memory friendly classification with the genome taxonomy database.” *Bioinformatics* 38: 5315-5316. https://doi.org/10.1093/bioinformatics/btac672

44. Parks, Donovan H, Maria Chuvochina, Christian Rinke, Aaron J Mussig, Pierre-Alain Chaumeil, Philip Hugenholtz. 2021. “GTDB: an ongoing census of bacterial and archaeal diversity through a phylogenetically consistent, rank normalized and complete genome-based taxonomy.” *Nucleic Acids Research* 50: D785-D794. https://doi.org/10.1093/nar/gkab776

45. Xu, Shuangbin, Zehan Dai, Pingfan Guo, Xiaocong Fu, Shanshan Liu, Lang Zhou, Wenli Tang, et al. 2021. “ggtreeExtra: Compact Visualization of Richly Annotated Phylogenetic Data.” *Molecular Biology and Evolution* 38: 4039-4042. https://doi.org/10.1093/molbev/msab166

46. Hyatt, Doug, Gwo-Liang Chen, Philip F. LoCascio, Miriam L. Land, Frank W. Larimer, Loren J. Hauser. 2010. “Prodigal: prokaryotic gene recognition and translation initiation site identification.” *BMC Bioinformatics* 11: 119. https://doi.org/10.1186/1471-2105-11-119

47. Aramaki, Takuya, Romain Blanc-Mathieu, Hisashi Endo, Koichi Ohkubo, Minoru Kanehisa, Susumu Goto, Hiroyuki Ogata. 2019. “KofamKOALA: KEGG Ortholog assignment based on profile HMM and adaptive score threshold.” *Bioinformatics* 36: 2251-2252. https://doi.org/10.1093/bioinformatics/btz859

48. Kanehisa, Minoru, Miho Furumichi, Mao Tanabe, Yoko Sato, Kanae Morishima. 2016. “KEGG: new perspectives on genomes, pathways, diseases and drugs.” *Nucleic Acids Research* 45: D353-D361. https://doi.org/10.1093/nar/gkw1092

49. Liu, Bo, Dandan Zheng, Qi Jin, Lihong Chen, Jian Yang. 2018. “VFDB 2019: a comparative pathogenomic platform with an interactive web interface.” *Nucleic Acids Research* 47: D687-D692. https://doi.org/10.1093/nar/gky1080

50. Johansson, Markus H K, Valeria Bortolaia, Supathep Tansirichaiya, Frank M Aarestrup, Adam P Roberts, Thomas N Petersen. 2020. “Detection of mobile genetic elements associated with antibiotic resistance in Salmonella enterica using a newly developed web tool: MobileElementFinder.” *Journal of Antimicrobial Chemotherapy* 76: 101-109. https://doi.org/10.1093/jac/dkaa390

51. Wang, Meng, Ying-Xian Goh, Cui Tai, Hui Wang, Zixin Deng, Hong-Yu Ou. 2022. “VRprofile2: detection of antibiotic resistance-associated mobilome in bacterial pathogens.” *Nucleic Acids Research* 50: W768-W773. https://doi.org/10.1093/nar/gkac321

52. Trappe, Kathrin, Tobias Marschall, Bernhard Y. Renard. 2016. “Detecting horizontal gene transfer by mapping sequencing reads across species boundaries.” *Bioinformatics* 32: i595-i604. https://doi.org/10.1093/bioinformatics/btw423

53. Song, Weizhi, Bernd Wemheuer, Shan Zhang, Kerrin Steensen, Torsten Thomas. 2019. “MetaCHIP: community-level horizontal gene transfer identification through the combination of best-match and phylogenetic approaches.” *Microbiome* 7: 36. https://doi.org/10.1186/s40168-019-0649-y

54. Guo, Jiarong, Ben Bolduc, Ahmed A. Zayed, Arvind Varsani, Guillermo Dominguez-Huerta, Tom O. Delmont, Akbar Adjie Pratama, et al. 2021. “VirSorter2: a multi-classifier, expert-guided approach to detect diverse DNA and RNA viruses.” *Microbiome* 9: 37. https://doi.org/10.1186/s40168-020-00990-y

55. Naccache, Samia N., Scot Federman, Narayanan Veeraraghavan, Matei Zaharia, Deanna Lee, Erik Samayoa, Jerome Bouquet, et al. 2014. “A cloud-compatible bioinformatics pipeline for ultrarapid pathogen identification from next-generation sequencing of clinical samples.” *Genome Research* 24: 1180-1192. https://doi.org/10.1101/gr.171934.113

56. Zaharia, Matei A., William J. Bolosky, Kristal Curtis, Armando Fox, David A. Patterson, Scott Shenker, Ion Stoica, Richard M. Karp, Taylor Sittler. 2011. “Faster and More Accurate Sequence Alignment with SNAP.” *ArXiv* abs/1111.5572,

57. Zhao, Yongan, Haixu Tang, Yuzhen Ye. 2011. “RAPSearch2: a fast and memory-efficient protein similarity search tool for next-generation sequencing data.” *Bioinformatics* 28: 125-126. https://doi.org/10.1093/bioinformatics/btr595

58. Greninger, Alexander L., Samia N. Naccache, Kevin Messacar, Anna Clayton, Guixia Yu, Sneha Somasekar, Scot Federman, et al. 2015. “A novel outbreak enterovirus D68 strain associated with acute flaccid myelitis cases in the USA (2012&#x2013;14): a retrospective cohort study.” *The Lancet Infectious Diseases* 15: 671-682. https://doi.org/10.1016/S1473-3099(15)70093-9

59. Jakobsen, Janus Christian, Christian Gluud, Jørn Wetterslev, Per Winkel. 2017. “When and how should multiple imputation be used for handling missing data in randomised clinical trials – a practical guide with flowcharts.” *BMC Medical Research Methodology* 17: 162. https://doi.org/10.1186/s12874-017-0442-1

60. Austin, Peter C., Ian R. White, Douglas S. Lee, Stef van Buuren. 2021. “Missing Data in Clinical Research: A Tutorial on Multiple Imputation.” *Canadian Journal of Cardiology* 37: 1322-1331. https://doi.org/10.1016/j.cjca.2020.11.010

61. Peng, Chen, Qiong Chen, Shangjin Tan, Xiaotao Shen, Chao Jiang. 2024. “Generalized reporter score-based enrichment analysis for omics data.” *Briefings in Bioinformatics* 25: https://doi.org/10.1093/bib/bbae116

62. Pariseau, Drew A., Brooke E. Ring, Saroj Khadka, Laura A. Mike. 2024. “Cultivation and Genomic DNA Extraction of Klebsiella pneumoniae.” *Current Protocols* 4: e932. https://doi.org/10.1002/cpz1.932

63. Chu, Hin, Jie Zhou, Bosco Ho-Yin Wong, Cun Li, Jasper Fuk-Woo Chan, Zhong-Shan Cheng, Dong Yang, et al. 2015. “Middle East Respiratory Syndrome Coronavirus Efficiently Infects Human Primary T Lymphocytes and Activates the Extrinsic and Intrinsic Apoptosis Pathways.” *The Journal of Infectious Diseases* 213: 904-914. https://doi.org/10.1093/infdis/jiv380

64. Acharya, Tankeshwar, Janelle Hare. 2022. Sabouraud Agar and Other Fungal Growth Media. *Laboratory Protocols in Fungal Biology: Current Methods in Fungal Biology* Springer International Publishing, 69-86. https://doi.org/10.1007/978-3-030-83749-5_2

65. Aufderheide, Michaela, Christine Förster, Morris Beshay, Detlev Branscheid, Makito Emura. 2016. “A new computer-controlled air–liquid interface cultivation system for the generation of differentiated cell cultures of the airway epithelium.” *Experimental and Toxicologic Pathology* 68: 77-87. https://doi.org/10.1016/j.etp.2015.10.001

66. Hillyer, Philippa, Rachel Shepard, Megan Uehling, Mina Krenz, Faruk Sheikh, Kalyn R. Thayer, Lei Huang, et al. 2018. “Differential Responses by Human Respiratory Epithelial Cell Lines to Respiratory Syncytial Virus Reflect Distinct Patterns of Infection Control.” *Journal of Virology* 92: 10.1128/jvi.02202-02217. https://doi.org/doi:10.1128/jvi.02202-17

67. Grubwieser, Philipp, Alexander Hoffmann, Richard Hilbe, Markus Seifert, Thomas Sonnweber, Nina Böck, Igor Theurl, Günter Weiss, Manfred Nairz. 2022. “Airway Epithelial Cells Differentially Adapt Their Iron Metabolism to Infection With Klebsiella pneumoniae and Escherichia coli In Vitro.” *Frontiers in cellular and infection microbiology* 12: https://doi.org/10.3389/fcimb.2022.875543

68. Schildgen, Verena, Stephanie Mai, Soumaya Khalfaoui, Jessica Lüsebrink, Monika Pieper, Ramona L. Tillmann, Michael Brockmann, Oliver Schildgen. 2014. “Pneumocystis jirovecii Can Be Productively Cultured in Differentiated CuFi-8 Airway Cells.” *mBio* 5: 10.1128/mbio.01186-01114. https://doi.org/doi:10.1128/mbio.01186-14

69. Rayner, Rachael E., Patrudu Makena, Gaddamanugu L. Prasad, Estelle Cormet-Boyaka. 2019. “Optimization of Normal Human Bronchial Epithelial (NHBE) Cell 3D Cultures for in vitro Lung Model Studies.” *Scientific Reports* 9: 500. https://doi.org/10.1038/s41598-018-36735-z

70. Kumar, Priti, Arvindhan Nagarajan, Pradeep D. Uchil. 2018. “Analysis of Cell Viability by the Lactate Dehydrogenase Assay.” *Cold Spring Harbor Protocols* 2018: pdb.prot095497. https://doi.org/10.1101/pdb.prot095497

71. van Tonder, Alet, Annie M. Joubert, A. Duncan Cromarty. 2015. “Limitations of the 3-(4,5-dimethylthiazol-2-yl)-2,5-diphenyl-2H-tetrazolium bromide (MTT) assay when compared to three commonly used cell enumeration assays.” *BMC Research Notes* 8: 47. https://doi.org/10.1186/s13104-015-1000-8

72. Del Valle, Diane Marie, Seunghee Kim-Schulze, Hsin-Hui Huang, Noam D. Beckmann, Sharon Nirenberg, Bo Wang, Yonit Lavin, et al. 2020. “An inflammatory cytokine signature predicts COVID-19 severity and survival.” *Nature Medicine* 26: 1636-1643. https://doi.org/10.1038/s41591-020-1051-9

73. Wang, Jinliang, Lei Shuai, Chong Wang, Renqiang Liu, Xijun He, Xianfeng Zhang, Ziruo Sun, et al. 2020. “Mouse-adapted SARS-CoV-2 replicates efficiently in the upper and lower respiratory tract of BALB/c and C57BL/6J mice*.” *Protein & Cell* 11: 776-782. https://doi.org/10.1007/s13238-020-00767-x

74. Bleul, Tim, Xinyu Zhuang, Antonia Hildebrand, Clemens Lange, Daniel Böhringer, Günther Schlunck, Thomas Reinhard, Thabo Lapp. 2020. “Different Innate Immune Responses in BALB/c and C57BL/6 Strains following Corneal Transplantation.” *Journal of Innate Immunity* 13: 49-59. https://doi.org/10.1159/000509716

75. Bao, Linlin, Wei Deng, Baoying Huang, Hong Gao, Jiangning Liu, Lili Ren, Qiang Wei, et al. 2020. “The pathogenicity of SARS-CoV-2 in hACE2 transgenic mice.” *Nature* 583: 830-833. https://doi.org/10.1038/s41586-020-2312-y

76. Ye, Zi-Wei, Chon Phin Ong, Kaiming Tang, Yilan Fan, Cuiting Luo, Runhong Zhou, Peng Luo, et al. 2022. “Intranasal administration of a single dose of a candidate live attenuated vaccine derived from an NSP16-deficient SARS-CoV-2 strain confers sterilizing immunity in animals.” *Cellular & Molecular Immunology* 19: 588-601. https://doi.org/10.1038/s41423-022-00855-4

77. Morales-Nebreda, Luisa, Monica Chi, Emilia Lecuona, Navdeep S. Chandel, Laura A. Dada, Karen Ridge, Saul Soberanes, et al. 2014. “Intratracheal administration of influenza virus is superior to intranasal administration as a model of acute lung injury.” *Journal of Virological Methods* 209: 116-120. https://doi.org/10.1016/j.jviromet.2014.09.004

78. Fouchier, Ron A. M., Thijs Kuiken, Martin Schutten, Geert van Amerongen, Gerard J. J. van Doornum, Bernadette G. van den Hoogen, Malik Peiris, Wilina Lim, Klaus Stöhr, Albert D. M. E. Osterhaus. 2003. “Koch's postulates fulfilled for SARS virus.” *Nature* 423: 240-240. https://doi.org/10.1038/423240a
